# LIPIDOMIC ANALYSIS OF GAIT SPEED IN THE LONG LIFE FAMILY STUDY

**DOI:** 10.1093/geroni/igac059.1558

**Published:** 2022-12-20

**Authors:** Adam Santanasto, Mary Fiestosa, Michael Lustgarten, Kaare Christensen, Gary Patti, Joseph Zmuda, Ryan Cvejkus, Mary Wojczynski

**Affiliations:** School of Public Health, University of Pittsburgh, Oakmont, Pennsylvania, United States; Washington University School of Medicine in St. Louis, St. Louis, Missouri, United States; Tufts University, Boston, Massachusetts, United States; Southern Denmark University, Odense, Syddanmark, Denmark; Washington University in St. Louis, St. Louis, Missouri, United States; School of Public Health, University of Pittsburgh, Oakmont, Pennsylvania, United States; School of Public Health, University of Pittsburgh, Oakmont, Pennsylvania, United States; Washington University in St. Louis, St. Louis, Missouri, United States

## Abstract

Gait speed is a predictor of overall health and mortality in older adults. Metabolomics may provide insights into biological mechanisms underlying gait speed. Herein, we examined the association between 193 lipid metabolites with gait speed in 1,717 adults (52.1% women) aged 82.0 ± 14.5. Lipidomic analysis was performed using liquid chromatography-mass spectrometry. Gait speed was measured over 4-meters and slowness was defined as <0.8m/s. Logistic regression, adjusted for age, sex, field center, height, fasting-duration and familial-relatedness, were used to examine the association between log-transformed metabolites with slowness. A false discovery rate (FDR) of p<0.05 was employed to account for multiple comparisons. Gait speed was 0.83 ± 0.32 and 53.4% had slowness. Three lipid metabolites were significantly associated with lower odds of slowness: an acylcarnitine, sphingomyelin and a ceramide non-hydroxy fatty acid-sphingosine. Our results potentially link lipids involved with mitochondrial beta-oxidation and nerve signal transduction to gait speed in older adults.

